# *In vivo* muscle morphology comparison in post-stroke survivors using ultrasonography and diffusion tensor imaging

**DOI:** 10.1038/s41598-019-47968-x

**Published:** 2019-08-14

**Authors:** Clara Körting, Marius Schlippe, Sven Petersson, Gaia Valentina Pennati, Olga Tarassova, Anton Arndt, Taija Finni, Kangqiao Zhao, Ruoli Wang

**Affiliations:** 1 Department of Mechanics, Royal Institute of Technology, Stockholm, Sweden; 2 Department of Medical Radiation Physics and Nuclear Medicine, Karolinska University Hospital, Stockholm, Sweden; 3 Department of Clinical Science Intervention and Technology, Karolinska Institutet, Stockholm, Sweden; 4 Karolinska Institutet, Department of Clinical Sciences, Danderyd Hospital, Division of Rehabilitation Medicine, Stockholm, Sweden; 5 The Swedish School of Sport and Health Sciences, Stockholm, Sweden; 6 Department of CLINTEC, Karolinska Institutet, Stockholm, Sweden; 7 Faculty of Sport and Health Sciences, University of Jyväskylä, Jyväskylä, Finland; 8 Department of Children’s and Women’s Health, Karolinska Institutet, Stockholm, Sweden; 9 KTH Biomex Center, Royal Institute of Technology, Stockholm, Sweden

**Keywords:** Skeletal muscle, Biomedical engineering

## Abstract

Skeletal muscle architecture significantly influences the performance capacity of a muscle. A DTI-based method has been recently considered as a new reference standard to validate measurement of muscle structure *in vivo*. This study sought to quantify muscle architecture parameters such as fascicle length (FL), pennation angle (PA) and muscle thickness (t_m_) in post-stroke patients using diffusion tensor imaging (DTI) and to quantitatively compare the differences with 2D ultrasonography (US) and DTI. Muscle fascicles were reconstructed to examine the anatomy of the medial gastrocnemius, posterior soleus and tibialis anterior in seven stroke survivors using US- and DTI-based techniques, respectively. By aligning the US and DTI coordinate system, DTI reconstructed muscle fascicles at the same scanning plane of the US data can be identified. The architecture parameters estimated based on two imaging modalities were further compared. Significant differences were observed for PA and t_m_ between two methods. Although mean FL was not significantly different, there were considerable intra-individual differences in FL and PA. On the individual level, parameters measured by US agreed poorly with those from DTI in both deep and superficial muscles. The significant differences in muscle parameters we observed suggested that the DTI-based method seems to be a better method to quantify muscle architecture parameters which can provide important information for treatment planning and to personalize a computational muscle model.

## Introduction

Skeletal muscles provide strength and protection by distributing loads and absorbing shocks. They perform both dynamic work, such as locomotion or positioning of the body segments in space, and static work, such as maintaining body posture or position^1^. Skeletal muscle architecture significantly influences the performance capacity of a muscle. Parameters that are often used to describe the muscle architecture are fascicle length (FL), pennation angle (PA), muscle thickness (t_m_) and physiological cross-sectional area (PCSA)^2^. It has also been shown that the macroscopic arrangement of muscle fibers can differ significantly between individuals e.g., stroke patients^3^, young/old individuals^4^, male/female^5^. *In vitro* measurements therefore limit the accuracy of muscle structure parameters when applied to individual persons or populations. *In vivo* measurements of muscle architecture can provide insights into the inter-individual variability of muscle function as well as reference data for the development of personalized muscle models for computational modeling. Conventionally, 2D ultrasound (US) and magnetic resonance imaging (MRI) are often used to quantify muscle structure *in vivo*. However, 2D US has limitations, since it only provides 2D images with a limited field of view, and a misalignment of the US transducer can lead to inaccurate measurements^6,7^. Moreover, assessing deeper muscles within the body such as the anterior soleus and tibialis posterior in the human lower limb can be difficult using US because image quality is often poor^5^. The anatomical MRI method can overcome some of the limitations of US^8^ but lacks the resolution to examine individual muscle fascicles.

Over the last decade, approaches using diffusion tensor imaging (DTI) to reconstruct muscle fascicles have evolved as an alternative reference standard^7,9,10^. Muscle DTI uses an MRI protocol to measure the diffusion of water molecules in the muscle tissue and can identify muscle fascicle direction because water molecules move primarily along the long axis of fascicles^11^. Studies have demonstrated that reconstructed fascicles from DTI aligned well with true muscles fascicles in rat^11^ and human skeletal muscles^10^. Heemskerk *et al*.^12^ and Bolsterlee *et al*.^10^ both evaluated the within-day and between-day repeatability of DTI tractography. In both studies the repeatability of measurements was shown to be acceptable. Bolsterlee *et al*. proposed a validation method based on DTI fascicle tracking compared with US measures of FL and PA with the same measures obtained using DTI in healthy gastrocnemius^10^. On average, US yielded slightly longer measurements of FL compared to DTI. Although mean measurements did not differ statistically, the intra-individual measures substantially varied. PA measured with US were found to be significantly smaller than the PA measured with DTI. To confirm the accuracy of muscle architecture parameters obtained using the DTI method, Bolsterlee *et al*. further compared the architecture parameters of soleus to those obtained based on cadaveric dissection^9^. Dissection measurements of FL were similar to those measured with DTI, but inconsistent PAs were observed in the posterior compartment of the soleus. Similar imaging techniques have also been applied to determine muscle volumes, lengths, cross sectional area and fiber orientations of the soleus in children with cerebral palsy (CP)^13^. Compared to able-bodied controls, cross sectional area perpendicular to the muscle fiber direction were reduced, but no differences were observed in fascicle length. The authors concluded that a better understanding of muscle architecture may motivate new therapies and help to predict their outcomes in children with CP^13^.

Stroke is a major global health problem; it is the second-most common cause of death and one of the leading causes of adult disability^14^. The skeletal muscle may undergo numerous structural and functional alterations following such an injury to the brain^15^. For instance, secondary changes after spasticity are commonly seen in many neurological disorders and impact approximately 30% of stroke patients^16^. Previous studies have indicated that spastic muscles may undergo morphological changes which alter the force generation capacity during movement and lead to an increased joint and muscle stiffness in hemiplegic persons^3,15^. Muscles around the ankle are often affected after stroke, and this contributes considerably to the disability^17^. The impaired ankle dorsiflexors, spastic plantarflexors, and associated foot drop make it difficult for the foot to clear from the ground during gait. In addition, weak plantarflexors also result in a reduced forward propulsion^18,19^. Shearwave elastography was applied on stroke survivors and persons with cerebral palsy in the biceps brachii, medial gastrocnemius and tibialis anterior^15,20,21^. Greater shearwave velocities were found in the affected muscles, which indicated higher muscle stiffness in biceps brachii and medial gastrocnemius on the paretic side^20^. However, no significant differences were observed in the tibialis anterior^15,20^. Therefore, authors concluded that muscles need to be evaluated individually to assess alterations^20^. Yang *et al*. assessed bilateral differences in t_m_, FL and PA of the gastrocnemius in stroke patients using US and found that t_m_ and PA were significantly larger but that FL was significantly smaller in the affected side^22^. Although US is widely used to quantify muscle architecture features, evidence of the validity of 2D US for measuring attributes such as FL and PA in human is limited^6^. The fidelity of these measurement in muscles with disrupted fascicles or fibrosis is uncertain, which likely occurs in post-stroke survivors^23^. The hyper-echoic appearance in muscle due to fat infiltration and increased fibrous tissue after neurological disorders, e.g., stroke, makes the muscle boundary and fascicle less distinct in US image. This feature leads to challenges and potential errors in muscle morphological parameter quantification in 2D US, especially in the deep muscles such as soleus. Moreover, 2D US imaging of muscle architectures has been suggested as a valuable tool to evaluate the functional improvement of muscles affected by neurological disease after an intervention program^24^, and the knowledge of specific parameters that are likely to be unreliable is essential for such clinical applications.

The aim of this study is two-fold: first, we seek to describe muscle architecture parameters such as FL, PA and t_m_ in medial gastrocnemius (GA), posterior soleus (PSO) and tibialis anterior (TA) in post-stroke patients *in vivo* using innovative DTI-based techniques; second, we seek to determine whether individual muscle morphological parameters in the GA, the PSO and the TA as identified through 2D US are similar to those obtained through DTI. Our findings may provide important information in rehabilitation strategy planning for stroke and shed light on the development of personalized computational muscle models. Based on the physics of ultrasonography and on the inherent differences in muscle echogenicity in neurologically pathological muscle tissue, our hypothesis was that the parameters that describe muscle architecture measured by the two methods would agree in superficial muscles but not in deep muscles.

## Methods

### Participants

In total, eleven hemiplegic post-stroke survivors were recruited from a local rehabilitation clinic (Department of Rehabilitation Medicine, Danderyd Hospital, Stockholm, Sweden). Four inclusion criteria were determined: (1) stroke >6 months prior to inclusion; (2) no anti-spastic treatment within three months; (3) eligibility for MR scanning, and (4) absence of other lower limb injuries or disorders. US measurement and DTI data acquisition occurred in two separate sessions but within one month. All participants gave written informed consent according to the Declaration of Helsinki. The study was approved by the Regional Ethics Committee, Stockholm, Sweden. All experiments were performed in accordance with relevant guidelines and regulations. Three participants dropped out after either the US or MRI measurements. Data from one participant were excluded because of the poor image quality, caused by motion artifacts. Therefore, experimental data from seven participants were included for further analysis (see Table 1 and Supplementary A Table A1).

**Table 1:** Characteristics of participants.

Subject	Gender	Age (Years)	Weight (Kg)	Height (cm)	BMI (kg/m^2^)	Lesion Type	Post Stroke Time (Months)
S1	M	50	85	169	29,8	Haemorrhagic	7
S2	M	31	79	178	24,9	Haemorrhagic	8
S3	F	71	88	182	26,6	Ischemic	29
S4	F	67	50	153	21,4	Haemorrhagic	60
S5	M	58	61	181	18,6	Haemorrhagic	84
S6	M	57	86	174	28,4	Haemorrhagic	37
S7	M	34	70	168	24,8	Haemorrhagic	25

### Data acquisition

**Figure 1:**
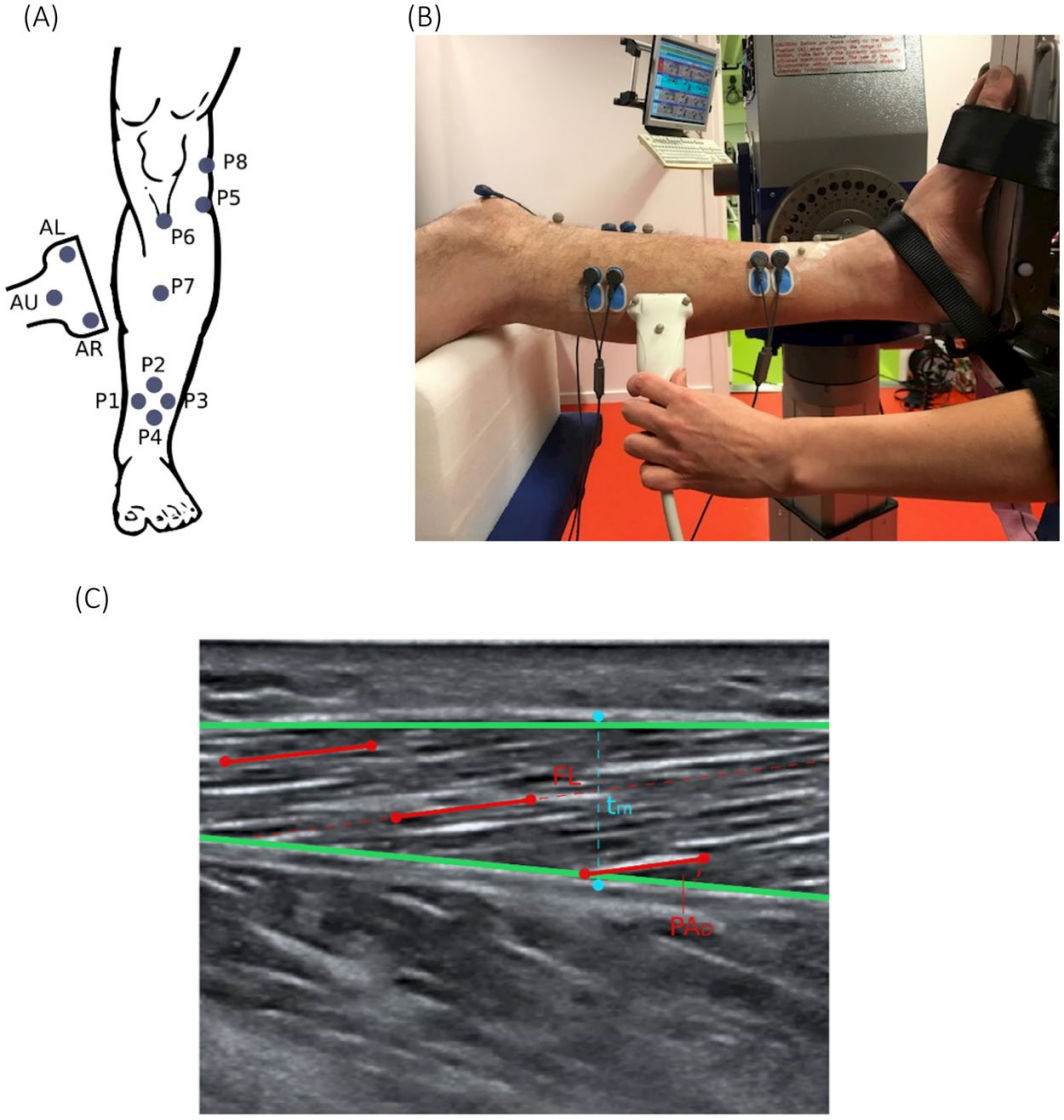
(**A**,**B**) Marker placement and experimental set-up during the ultrasound measurement. Markers P1, P2, P3 and P4 were fixed on to a rectangle silicon pad and attached to the lower limb. Marker P5 and P6 were placed on the head of the fibula and the tuberosity of the tibia, respectively. Marker P7 was placed 10 cm distal to P6 along the tibia bone. P8 was placed on the medial side of the epicondyle. Marker P4, P7 and P6 were carefully adjusted in order to align in a straight line. AL, AR and AU were the markers placed on the US transducer, where AL and AR defined the acquisition window of the transducer. (**C**) Example of the measurement of the parameters pennation angle (PA), muscle thickness (t_m_) and fascicle length (FL) of the tibialis anterior muscle in an US image using MATLAB (green lines: deep and superficial aponeurosis, red lines: selected fascicles). The muscle thickness was identified by setting two points (blue markers) marking each of the aponeuroses.

During US data acquisition, participants were seated in a comfortable semi-upright position and were instructed to remain relaxed and static during the measurement with their knee flexed at 30° and ankle at 10° plantarflexion. Their foot was fixated to a foot plate connected to a dynamometer (IsoMed 2000, D&R GmbH, Hemau, Germany). The US data were collected using an M9 Mindray ultrasonography system (Mindray M9, Shenzhen, China) with a 38 mm wide linear transducer (6–14 MHz). The imaging investigator, a licensed physician, received sonography training in a separate ultrasonographic clinic before taking the images for this study. The same investigator imaged all participants. In order to determine the coordinate system of US measurement (in a leg-based reference frame), a motion capture system (Qualisys, Gothenburg, Sweden) in combination with the US system was utilized. Eight reflective markers were placed on anatomical landmarks of the participant’s affected leg and three markers were placed on the US transducer (Fig. 1A,B). The marker locations were later used to determine the location and orientation of the US image with respect to a leg-based frame.

During DTI data acquisition, all patients were scanned bilaterally using a 3T MRI scanner (Siemens Trio, Siemens Medical Solution, Erlangen, Germany) while lying in a supine position where the thigh was resting on a wedge and the foot strapped in a footplate. The configuration of the knee and ankle angles was set to be identical to that in the US measurement. Prior to scanning, eight markers were also placed on the landmarks of the participant’s affected side by the same instructor (in the same location as in the US measurement), which were visible in T1-weighted MR images. The T1-weighted images were obtained with the following settings: TSE sequence, TR/TE 605 ms/23 ms, field of view (FOV) 430 mm, acquisition matrix 512 × 299 pixel, voxel size 0.84 × 0.84 × 5 mm and scan time 101 s. The settings of the DTI images were: EPI sequence, FOV 350 mm, acquisition matrix 140 × 140 pixel, voxel size 2.5 × 2.5 × 2.5 mm, 20 DTI gradient directions, number of signal averages 4, b = 500 s/mm^2^ (B0 image with b = 0 s/mm^2^, with 2 signal averages), and scan time 520 s.

### Ultrasound fascicle reconstruction

US images were analyzed using a custom-written MATLAB program (version: R2017b) that was, similar to previous reports^25^. The deep and superficial aponeuroses of each respective muscle were firstly identified. Three muscle fascicles (for as long as the fascicles were clearly visible) in the proximal, distal, and intermediate part of the muscle were then selected in the image. Finally, the t_m_ was identified as the distance between the superficial and deep aponeuroses (Fig. 1C). The deep and superficial PAs were identified as the angles formed between the fascicles and the line of the proximal and distal aponeuroses. The reported PA resulted from calculating the average of the deep and superficial PAs. The FL was calculated as the straight-line distance by dividing the t_m_ by the sine of the deep PA.

### DTI fascicle reconstruction

**Figure 2:**
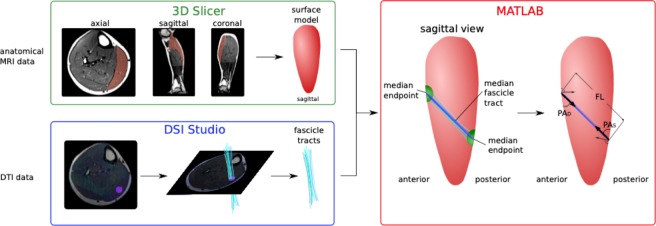
Procedure of fascicle tracking based on MRI and DTI data. The anatomical MRI images were manually segmented, and a surface model of each muscle was created. In parallel, the DTI data were reconstructed within DSI studio and 100 fascicle tracts (in light blue) were tracked based on a small seeding area (in purple). The tracts were imported into MATLAB (^5^,version: R2017b) together with the surface models. The median end points of all 100 tracts were calculated and translated towards the muscle surface in both directions. When the surface was intersected, the FL was obtained by calculating the distance between both median endpoints. The superficial and deep pennation angle PA_S_ and PA_D_, represent the angle of the median tract to the surface of the muscle volume in both endpoints.

The reconstruction of muscle fascicles and calculation of the DTI-based muscle parameters were modified based on a previously reported method^7^. Briefly, it consisted of four steps: (1) segmentation of muscles based on T1-weighted images; (2) denoising of DTI data using a Local Principle Component Analysis filter^26^; (3) fascicle tractography using DSI studio to generate 100 tracts through a small region within the target muscle [settings: 0.7 < fractional anisotropy <0.1, maximum angle between tract segments 10°, 20 mm ≤ tract length ≤ 200 mm; step size = 1.0 mm)]^27^; and (4) muscle parameter identification (Fig. 2). The muscle segmentation for this project was carried out manually for each patient using the open-source platform 3D Slicer (on average 80 slices per scan with a spacing of 5 mm between slices). 3D Slicer was used to calculate 3D surface models from the segmented image data which is done by using a pipeline of algorithms: creating a binary label map from the segmentation, generating a marching cubes model, and running triangle reduction and triangle smoothing using the smoothing factor of 0.5. To ensure correct segmentations, the segmentations were verified by an independent expert. To calculate fascicle parameters, the tracts were overlain over the surface mesh calculated from the muscle segmentation. To reconstruct the end points of the fiber tracts, the median x-, y- and z-coordinates of the 100 endpoints at either end of the tracts were determined and towards the muscle surface along the line connecting both median endpoints until the surface was intersected. The FL was obtained by calculating the Euclidean distance between the two endpoints on the superficial and deep aponeurosis, and the PA was calculated as the average angle of the median tract to the normal vectors of all of the surface triangles inside a radius of 5 mm around the respective endpoint. The reported PA resulted from calculating the average of the deep and superficial PAs. The t_m_ was measured in the T1-weighted MRI images within 3D slicer by marking each of the aponeuroses manually.

### Alignment of ultrasound and MRI coordinate system

**Figure 3:**
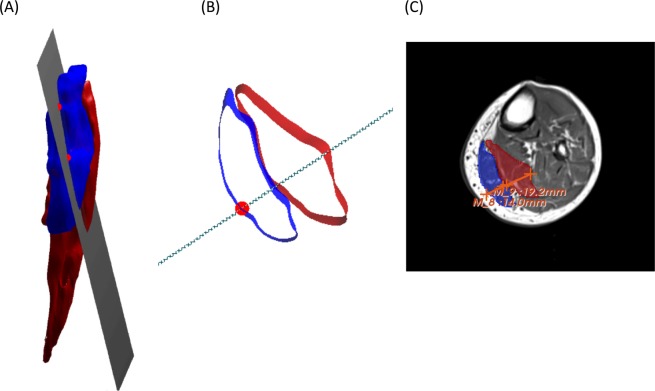
(**A**) Visualization of the muscle volumes of the medial gastrocnemius (blue) and medial-posterior soleus (red) with the transformed US plane cutting through the muscle volumes. The red markers specifying the width of the US transducer. (**B**) The visualization of the US plane intersection with the muscle volumes within the field of view of the US transducer (red markers). (**C**) The corresponding MRI image of the muscles. The thickness measurements were done within the 3D slicer software using the provided ruler tool^24^.

In order to locate DTI tracks within the muscle at the same scanning plane of the US data, the US coordinate system and the MRI coordinate system were aligned. The alignment was based on a four-marker cluster: P1, P2, P3 and P4 on the tibia (Fig. 1A,B). The additional markers, AL and AR, on the US transducer were used to define the acquisition window of the transducer. The coordinate system alignment was performed by transforming the coordinates of the markers AL, AR and AU from the US global coordinate system to the MR global coordinate system and creating a scanning plane based on the three markers on the probe. The created plane was displayed together with the segmented muscle volumes and the marker positions AR and AL indicating the position of the transducer on the patient’s skin. For comparing US and DTI measurements, fiber tracking was performed at the site of the intersection of the US plane with the segmented muscle surfaces (Fig. 3A).

### Data analysis

FL, PA and t_m_ were obtained for each participant in three muscle compartments of the affected limb (GA, PSO and TA) using two methods (i.e., US and DTI). For muscle parameters based on US data, three muscle fascicles in the proximal, distal and intermediate part of the muscle were then selected in the image and FL and PA were averaged for each muscle. For muscle parameters based on DTI data, three seed regions were chosen, and the resulting FL and PA values were averaged for each muscle. t_m_ was measured in the MRI images manually in three slices and averaged (Fig. 3B,C). Statistical analyses were performed using IBM SPSS Statistics (IBM Corp., Armonk, NY, USA). Descriptive statistics were reported as median and range values. Wilcoxon signed-rank test was used to investigate the differences between muscles parameters identified by DTI- and US-based methods, respectively. Differences were considered statistically significant when *p* ≤ 0.05. A mean (standard deviation) within-subject absolute differences by subtracting matching pairs of US and DTI measurements were also calculated. In order to account for the effects of different participant height, statistical analysis was also performed on the fascicle length and thickness normalized by height.

## Results

### 3D muscle morphological parameters of the affected-side in post-stroke participants

**Figure 4:**
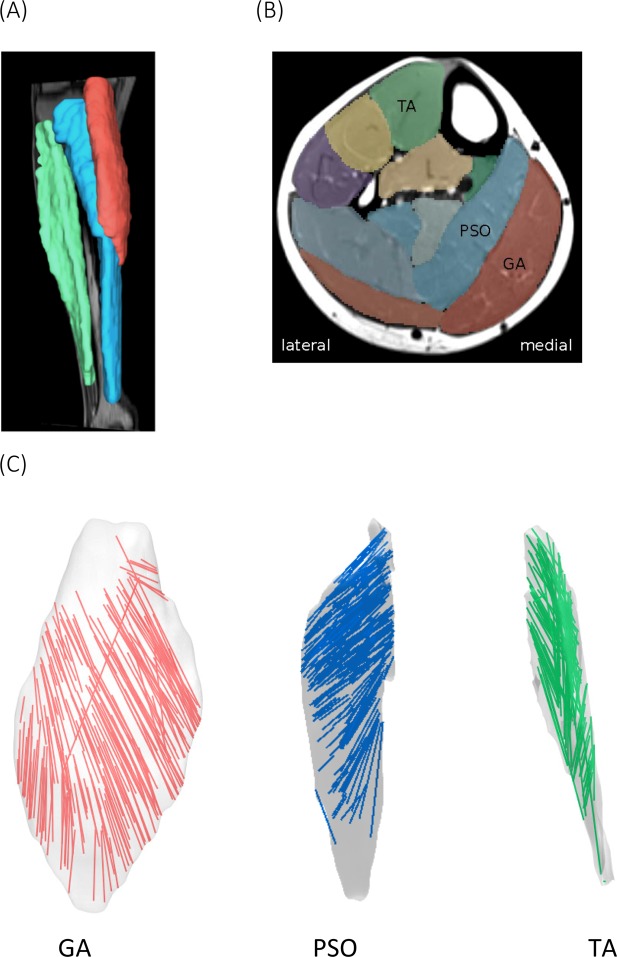
(**A**) The surface model of medial gastrocnemius (GA, in red), medial-posterior soleus (PSO, in blue) and tibialis anterior (TA, in green) were created based on anatomical MR images. (**B**) The transverse view of the anatomical MR slice approximately midway between the ankle and knee. (**C**) 3D reconstructed fascicle tracks of GA, PSO and TA based on DTI data.

**Figure 5:**
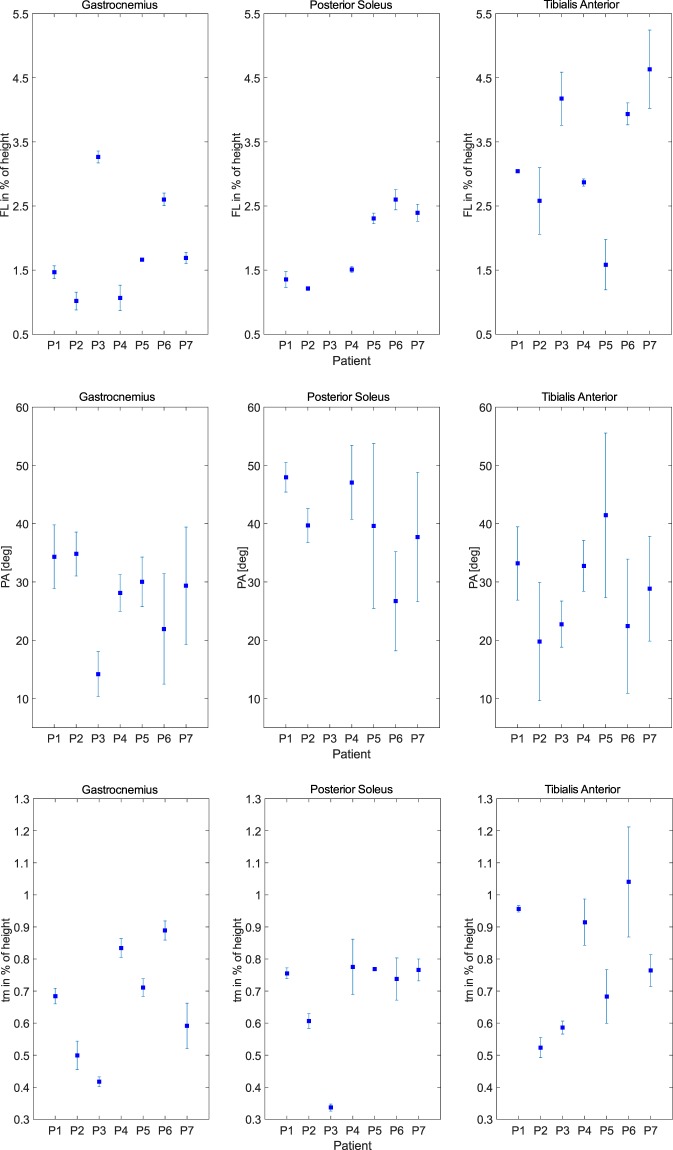
Individual muscle architecture parameters estimated using DTI-based method (FL: fascicle length, t_m_: muscle thickness, PA: pennation angle). FL and t_m_ were normalized by individual’s height.

Three distinct compartments can be clearly identified from the MRI and DTI scans of all post-stroke survivors, except the PSO from one participant, which was excluded from further analysis because the tracts within a bundle did not show a consistent pattern. Reconstructed 3D muscle fascicles from an example participant were illustrated in Fig. 4 and showed a unipennate structure in the GA and PSO and a more complexed fascicle orientations in TA. Large within-subject differences were observed in the PAs in all three muscles (Fig. 5). Compared to GA and PSO, considerable intra-subject differences in FL were found in TA (Table 2). Average FL ranged from 16 mm to 59 mm in GA, 22 mm to 45 mm in PSO, and from 29 mm to 78 mm in TA respectively. Average PA ranged from 14° to 35° in GA, from 27° to 48° in PSO and from 20° to 41° in TA, respectively.

**Table 2:** Comparison of measurements of the medial gastrocnemius (GA), posterior soleus (PSO) and tibialis anterior (TA) of the affected leg of the stroke survivors using ultrasound- (US) and diffusion tensor imaging- (DTI) based methods, expressed as median (Min, Max) of the group.

Muscle	Fasicle Length (FL) (mm)		Pennation Angle (PA) (°)		Muscle Thickness (t_m_) (mm)	
	US	DTI	US	DTI	US	DTI
GA	34.1 (23.8, 42.8)	28.4 (16.3, 59.4)	**13.4** (7.8, 16.1)	**29.3**a (14.2, 34.8)	**9.0** (7.6, 15.3)	**11.6**^**c**^ (7.6, 15.5)
PSO	34.0 (32.1, 51.2)	31.7 (21.6, 45.2)	**20.1** (12.8, 27.2)	**39.6**^**b**^ (26.7, 48.0)	11.3 (6.5, 15.0)	12.8 (10.8, 13.9)
TA	45.0 (33.4, 75.5)	51.4 (28.7, 77.9)	**7.4** (5.2, 15.3)	**32.8**^**b**^ (22.4, 41.3)	**8.2** (5.4, 12.4)	**12.8**^a^ (9.3, 18.1)

Bold-faced values indicated that the significant differences were observed between two methods. ^a^*p* = 0.02. ^b^*p* = 0.03. ^c^*p* = 0.05.

### Comparison of muscle morphological parameters using US and DTI methods

**Figure 6:**
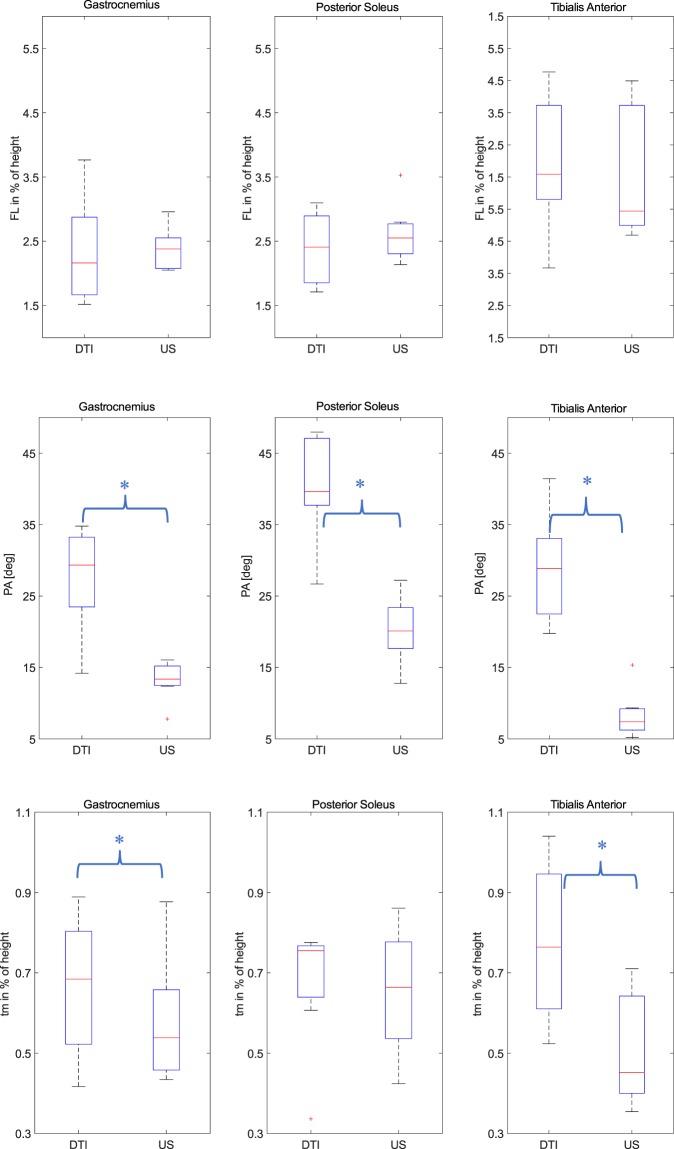
Comparison of muscle architecture parameters of the post-stroke subjects (affected-side) using DTI fascicle tracking and US measurements using box plot (FL: fascicle length, t_m_: muscle thickness, PA: pennation angle). The ‘+’ symbol denotes the outliners. The asterisk denotes significant differences between two methods. FL and t_m_ were normalized by individual’s height.

Almost all cases (approximately 86%) an intercepting plane of the US image through the muscle volume could be calculated and fascicle tracking as well as muscle thickness measurements could be carried out in the same location as the measurements performed in the US data. Compared to the DTI-based method, significantly smaller PAs were observed in all three muscles (GA: *p* = 0.02, PSO: *p* = 0.03, TA: *p* = 0.03) using US-based measurements (Fig. 6 and Table 2).The t_m_ was also found to be significantly smaller in medial GA (*p* = 0.05) and TA (*p* = 0.02). No significant differences were found in reconstructed FL between the two methods. However, a large standard deviation was observed for within-subject differences between US and DTI measurements in FL in all three muscles (Table 3, GA: 10.82 mm, PSO: 8.50 mm and TA: 15.47 mm). Comparable observations were found by performing analysis using normalized FL and t_m_.

**Table 3:** Mean within-subject absolute differences between ultrasound and DTI measurements in the medial gastrocnemius (GA), posterior soleus (PSO) and tibialis anterior (TA) parameters.

Muscle	Mean paired absolute difference (standard deviation)		
	Ultrasound - DTI		
	Fasicle Length (FL) (mm)	Pennation Angle (PA) (°)	Muscle Thickness (t_m_) (mm)
GA	10.6 (10.8)	14.3 (8.4)	1.4 (1.8)
PSO	11.6 (8.5)	19.6 (10.7)	2.1 (1.7)
TA	22.7 (15.5)	20.4 (7.8)	4.6 (2.8)

## Discussion

This study used both US- and DTI-based techniques to examine the muscle anatomy of the lower limb of stroke survivors in *vivo*. Significant differences were observed in both PAs and t_m_ in all three muscles between the two methods. Although the measured FL was not significantly different between the two methods, a large standard deviation was observed for the within-subject differences in both superficial and deep muscles. These findings only partially support our hypothesis. To our knowledge, this is the first study comparing these two imaging methods in measuring muscle architecture parameters in post-stroke participants.

Muscle architecture is one determinant of a muscle’s force-generating capacity. For instance, muscle FL is the primary determinant of muscle excursion, where shorter muscle fascicles have a smaller range of excursion that can develop force and power^28^. Structure changes such as FL and PAs have been commonly investigated using ultrasonography. However, large variability was found in the reported data in post-stroke participants due to several factors such as potential errors in the US measurement, the nature of the inhomogeneity of the patient group, and different joint configurations in the measurement. The previously reported mean value of the FL and PA of the paretic GA after stroke in a fully extended knee and a neutral ankle position ranged from 36 mm to 51 mm and 16° to 25°, respectively^29–31^. These previously reported the FL of GA are slightly longer than those found in the present study. The discrepancy could be due to the flexed-knee position we adopted during the measurement. Very few studies have reported the muscle structure parameters of the PSO and TA using ultrasonography. Ramsay *et al*. reported mean FL and PA of 39.7 ± 10.4 mm and 18.0° ± 3.4° in the PSO^29^, and 26.38 ± 4.2 mm and 13.4° ± 2.7° in the TA, respectively^32^. Our fascicle length of the PSO was within the range of reported values. However, the fascicle length of the TA was longer. The PAs of all muscles fell within the range of the reported values. DTI-based muscle architecture measures have been introduced in recent years and, can provide a three-dimensional measurement of the muscle architecture. However, the methodology has only been applied in healthy persons, and muscle structure parameters have only been reported in the GA and PSO. Compared to the reported data from healthy participants^9,10^, the FLs in our post-stroke survivors were shorter and the PAs were larger in the GA, though the differences in the PSO were not as distinct as the other two muscles.

Accurate assessment of changes in the muscle architecture can provide crucial information for planning treatment modalities such as stretching and orthotic management^28^. The comparison between US and DTI parameters showed a sufficient agreement of mean values for FL in three muscles, which agreed with a previous study comparing US and DTI FL estimation in the GA in healthy participants^9^. Similar to the abovementioned study, we also observed a large deviation of the within-subject differences between US and DTI FLs in the stroke survivors (Table 3), which warrant caution when using US-based measurements to guide treatment planning and personalize FL in a musculoskeletal model. Muscle thickness is considered to be strongly correlated with muscle mass and strength^33,34^; thus, it is often assessed by US in a clinical setting. We found significantly larger t_m_ estimation in GA and TA using DTI, but no differences were observed in PSO. This is likely due to a compression of the muscle by the pressure applied by the US transducer on the superficial muscles. No data have been published that compare US and DTI-based muscle thickness estimations comparison either in healthy or in post-stroke participants. Compared to GA and PSO, the TA thickness showed a slightly higher mean within-subject difference of 4.6 mm. Although the TA is anatomically divided into two compartments by a medial aponeurosis, the visibility of the aponeurosis varied in patients from the US images, affecting the accuracy in calculating the thickness of the tibialis anterior. In our study, mean PAs measured with US were significantly smaller than angles measured with DTI in post-stroke survivors. However, the differences in PA between US and DTI are larger than the differences reported by Bolsterlee *et al*.^9^ and Bérnard *et al*. in healthy GA^35^. This could be due to several reasons. For example, the muscle could be deformed by the compression from the US probe. Rheological muscle property changes such as fibrosis and atrophy are common in the spastic muscle^36^. In practice, increased pressure was sometimes necessary to apply in the probe in order to achieve a better visibility of the fascicles. In addition, unlike the healthy participants, joint alignment was difficult in some patients due to spasticity or pain, which may have caused some inconsistencies in joint alignment between the US and DTI measurements, even though the identical joint alignment set-up was adopted in both measurements.

In clinical practice, the rehabilitation plan after stroke is usually selected based on subjective clinical experience rather than on an objective prediction of post-treatment function developed based on individual data. Personalized computational models of the neuromusculoskeletal system could therefore facilitate the objective prediction of patient-specific functional outcome for different treatment designs. Using DTI-based muscle architecture measurements, the complex muscle structure of different compartments and locations can be discerned *in vivo*. Based on our comparison in post-stroke survivors, FL and PA are more sensitive to the measurement method, and TA is more sensitive than the other two plantarflexors.

There are several limitations in the current study. First, the signal intensity of the posterior part of the leg was lower than the anterior part of the leg in some participants. In the future, using an alternative coil set-up could improve the image intensity and lead to a better quality of the DTI data. Second, the accuracy of the muscle parameter estimation could be improved by using more than three seed points for each muscle compartment or by tracking fascicles within the whole muscle volume. Third, calculating FL as Euclidean distance between the two endpoints might underestimate the actual tracts. Using a polynomial curve fitting to account for the curvature of the fascicles may improve the accuracy in FL estimation. Fourth, further investigation including an age- and gender-matched control group would be valuable to provide additional information of muscle morphological alternations after stroke. However, this was out of the scope of the current study. Finally, the sample size of the patients is small, and only the affected side was measured using US due to the time constraint of the patient. Future studies with larger cohorts and multiple measurements will improve the reliability and generalization of our findings to the entire stroke population.

## Conclusions

Using a novel DTI-based imaging method, 3D muscle architecture parameters of GA, PSO and TA including FL, PA and t_m_ were described for the first time in stroke survivors *in vivo*. By aligning US and MR coordinate system, the muscle parameters obtained using DTI-based method were further compared with parameters obtained using a US-based method. DTI and US measurements of FL were similar on a group level, but significantly smaller t_m_ and PA estimation were observed in US. Moreover, a large standard deviation for the within-subject differences between the two methods was found with respect to FL and PA. Compared to 2D US, DTI-based method seems to be a better option to provide important reference information for treatment planning as well as to personalize a musculoskeletal model which helps to improve the fidelity of modeling prediction of rehabilitation outcomes after stroke. Even though using a 2D US to quantify muscle architecture parameters is much more feasible generally, caution should be taken before personalizing musculoskeletal models in a patient population of stroke survivors. On the individual level, parameters measured by US agreed poorly with those from DTI in both deep and superficial muscles. A consideration of the required level of reliability for each parameter should be taken in modelling.

## Supplementary information

Supplementary A
