# Supplementary material for: In vivo muscle morphology comparison in post-stroke survivors using ultrasonography and diffusion tensor imaging
Source: Sci Rep. 2019 Aug 14;9:11836. doi: 10.1038/s41598-019-47968-x (PMC6694129; doi:10.1038/s41598-019-47968-x)
Supplement: Supplementary file 1 — Supplementary A [file 41598_2019_47968_MOESM1_ESM.pdf]

Supplementary A

Title: *In vivo* muscle morphology comparison in post-stroke survivors using ultrasonography and diffusion tensor imaging

Clara Körting, Marius Schlippe, Sven Petersson, Gaia Valentina Pennati, Olga Tarassova, Anton Arndt, Taija Finni, Kangqiao Zhao, Ruoli Wang

Table A1 Background information of participants. The positive angle indicated dorsiflexion and negative angle indicated plantarflexion. GA: medial gastrocnemius, SO: soleus, TA: tibialis anterior

**Subject**	**Passive Range of Motion (degree)**	**Walking Aids**	**Spasticity (GA, SO, TA)**	**Clonus (GAS, SO, TA)**	**Previous botulinum toxin injection (GA, SO, TA)**
S1	45 [-40, 5]	orthoses	no	no	SO
S2	45 [-40, 5]	no	GA, SO	GA, SO	SO
S3	55 [-50, 5]	cane	GA	GA	GA
S4	40 [-45, -5]	wheel chair/orthoses	GA, SO	GA, SO	no
S5	45 [-50, -5]	wheel chair	NA	NA	NA
S6	45 [-40, 5]	orthoses	GA, SO	GA	no
S7	30 [-35, -5]	orthoses/cane	GA	GA	no

Additional analysis

Spearman's rank order correlation test was used to investigate the correlation of body mass index (BMI) and stroke duration with muscle parameter in GA, PSO and TA, respectively (Table A2). We also tested correlation between the stroke duration and within-subject differences observed in muscle parameters between US and DTI measurements (Table A3). Correlations were found weak to moderate, but not significant. A significantly negative and high correlation was observed between BMI and mean paired absolute differences in muscle thickness of medial gastrocnemius. Due to small sample size, the correlation needs to be interpreted with cautions.

Table A2 The correlation between muscle morphological parameters estimated using DTI-based techniques and body mass index (BMI) and stroke duration for medical gastrocnemius (GA), posterior soleus (PSO) and tibialis anterior (TA) were investigated using Spearman's rank order test. FL: fascicle length, PA: pennation angle, MT: muscle thickness

**Correlation Coefficient (r_s)**	**GA**			**PSO**			**TA**		
	**FL**	**PA**	**MT**	**FL**	**PA**	**MT**	**FL**	**PA**	**MT**
**BMI**	0.25	-0.07	-0.14	0.26	-0.14	-0.35	0.42	-0.36	0.39
**Stroke Duration**	0.21	-0.46	0.57	0.66	-0.37	0.35	-0.32	-0.18	0.07

Table A3 The correlation between within-subject differences observed in muscle parameters between US and DTI measurements and body mass index (BMI) and stroke duration for medical gastrocnemius (GA), posterior soleus (PSO) and tibialis anterior (TA) were investigated using Spearman's rank order test. FL: fascicle length, PA: pennation angle, MT: muscle thickness

**Correlation Coefficient (r_s)**	**Mean paired absolute difference (US-DTI)**								
	**FL**			**PA**			**MT**		
	**GA**	**PSO**	**TA**	**GA**	**PSO**	**TA**	**GA**	**PSO**	**TA**
**BMI**	0.29	0.23	-0.18	0.21	0.26	-0.61	**-0.93**	0.03	0.14
**Stroke Duration**	-0.32	-0.54	0.64	-0.39	-0.20	0.39	0.61	0.14	0.29

Significant correlation was denoted in bold, P< 0.01
